# Spontaneous Pneumomediastinum: A Diagnostic Challenge in a Patient Presenting With Excessive Vomiting and Neck Swelling

**DOI:** 10.7759/cureus.68396

**Published:** 2024-09-01

**Authors:** Nuh Umar, Hannah Charlotte Copley, Dipankar Chattopadhyay

**Affiliations:** 1 General Surgery, Bedfordshire Hospitals NHS Foundation Trust, Bedford, GBR

**Keywords:** vomiting, subcutaneous emphysema, boerhaave syndrome, hamman’s syndrome, spontaneous pneumomediastinum (spm)

## Abstract

Hamman’s syndrome, or spontaneous pneumomediastinum, refers to free air in the mediastinum without an apparent cause and typically affects young people. This case report describes a 33-year-old man who presented with neck swelling following excessive vomiting due to alcohol consumption. Clinical examination revealed subcutaneous emphysema of the neck, and imaging confirmed pneumomediastinum. Initial suspicion of Boerhaave syndrome led to aggressive supportive management, but further imaging ruled out oesophageal perforation, confirming Hamman’s syndrome. The patient was treated conservatively and discharged after a successful trial of a light diet. This case highlights the diagnostic challenges of Hamman’s syndrome, given that its symptoms overlap with more serious conditions like Boerhaave syndrome. Prompt recognition and appropriate conservative management are essential for favourable outcomes, emphasizing the benign and self-limiting nature of Hamman’s syndrome.

## Introduction

Spontaneous pneumomediastinum is defined as free air in the mediastinum without any apparent cause after more serious causes such as iatrogenic injuries or infections by gas-producing bacteria have been excluded [1]. This condition was first officially described by Dr. Louis Hamman in 1939 and hence is also known as Hamman’s syndrome [2]. It has been reported in many patient populations, most commonly in the 14-35 years age group and runs a benign course [3,4]. A possible pathophysiological mechanism for this condition was described by Macklin and Macklin [5], who proposed that a sudden rise in intrathoracic pressure results in rupture of terminal alveoli, followed by tracking of air from interstitium up to mediastinum due to pressure differential.

The primary reported aetiologies contributing to spontaneous pneumomediastinum include strenuous physical exertion, labour, pulmonary barotrauma, severe paroxysmal coughing, vomiting, and asthma. Some authors have highlighted narcotic consumption as an important instigator of spontaneous pneumomediastinum too [6]. The common clinical manifestations of Hamman's syndrome include chest discomfort, prolonged coughing, throat irritation, difficulty in swallowing, and shortness of breath. Notably, subcutaneous emphysema of the neck and face emerges as the prevailing physical examination finding, although its frequency varies in the literature from 50% to 70% [4,7].

Due to the constellation of signs and symptoms being non-specific, it is not uncommon for the diagnosis to be missed or delayed [8]. Many of the risk factors and symptoms of Hamman’s syndrome overlap with oesophageal rupture, also known as Boerhaave syndrome [9]. This case report discusses the difficulty in reaching a diagnosis of Hamman’s syndrome due to a mistaken initial diagnosis of Boerhaave syndrome. Hamman’s is usually diagnosed through clinical examination along with a plain chest radiograph. In some cases, a CT scan is necessary to diagnose [10]. The mainstay of treatment includes bed rest, monitoring, analgesia, and oxygen therapy [7]. Some patients may derive benefit from antibiotics should there be elevated inflammatory markers [11].

## Case presentation

A 33-year-old man presented to our district general hospital accident and emergency department with a primary presenting complaint of neck swelling. This had been noted a few hours prior to admission following a 16-hour episode of excessive vomiting secondary to alcohol excess (consumption of 19 units of alcohol the preceding evening). Other symptoms disclosed included a sore throat, widespread feeling of crackling throughout the neck bilaterally, and the absence of chest pain or shortness of breath. There were no reported issues with eating and drinking. On examination, the patient was alert and oriented with a Glasgow Coma Scale (GCS) score of 15 and with vital signs within normal ranges. There was an asymmetrical neck swelling (greater on the left side), with normal skin appearances. On palpation, there was extensive subcutaneous emphysema of the neck, with no facial or chest involvement. His past surgical history included repair of undescended testes in childhood, and he typically drank four units of alcohol a week and about five cigarettes a day, with occasional use of cannabis but no other illegal drugs. He was otherwise fit and healthy, with no regular medications or known allergies.

**Figure 1:**
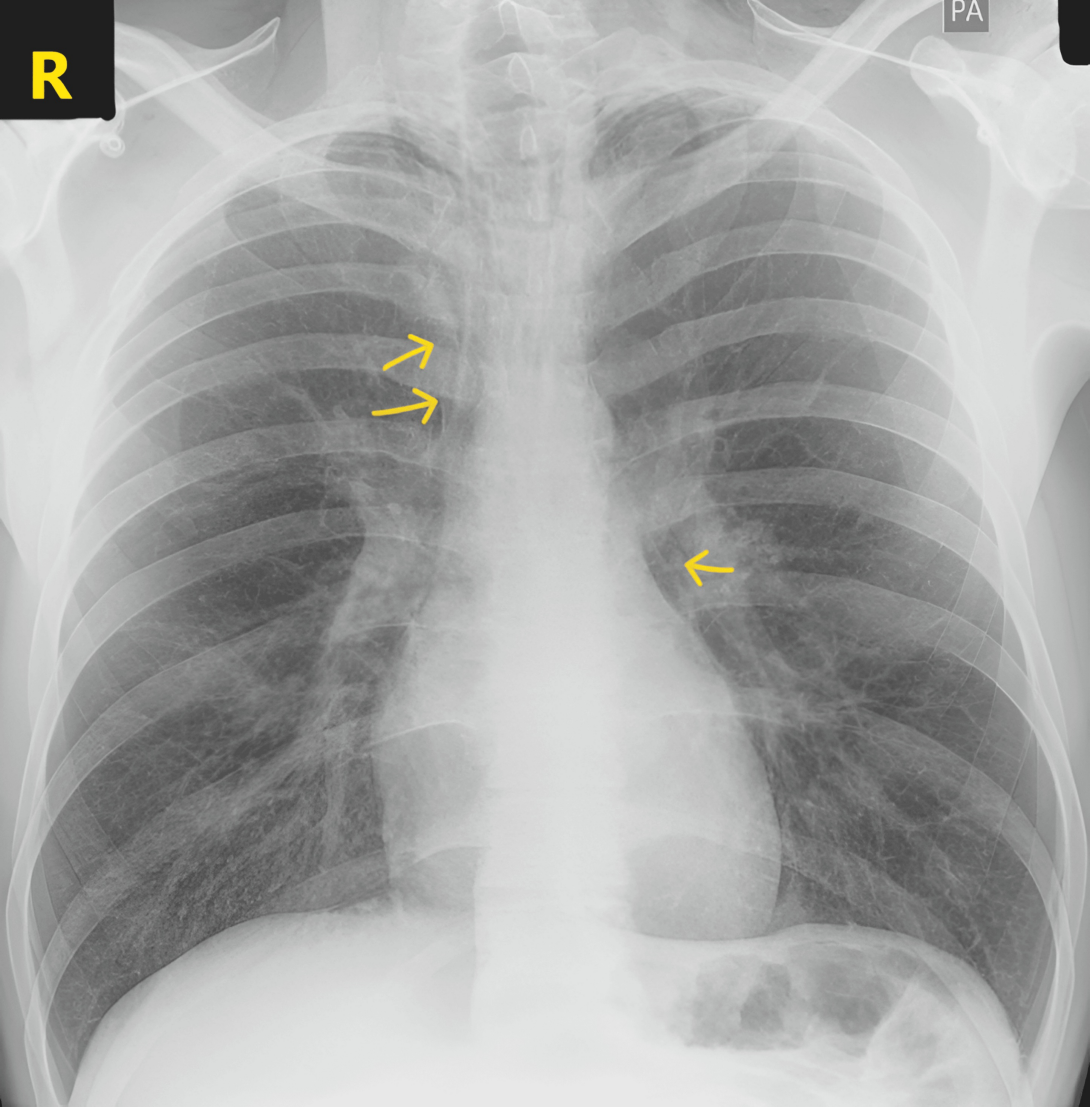
Chest X-ray (posteroanterior view) showing pneumomediastinum (yellow arrow).

**Figure 2:**
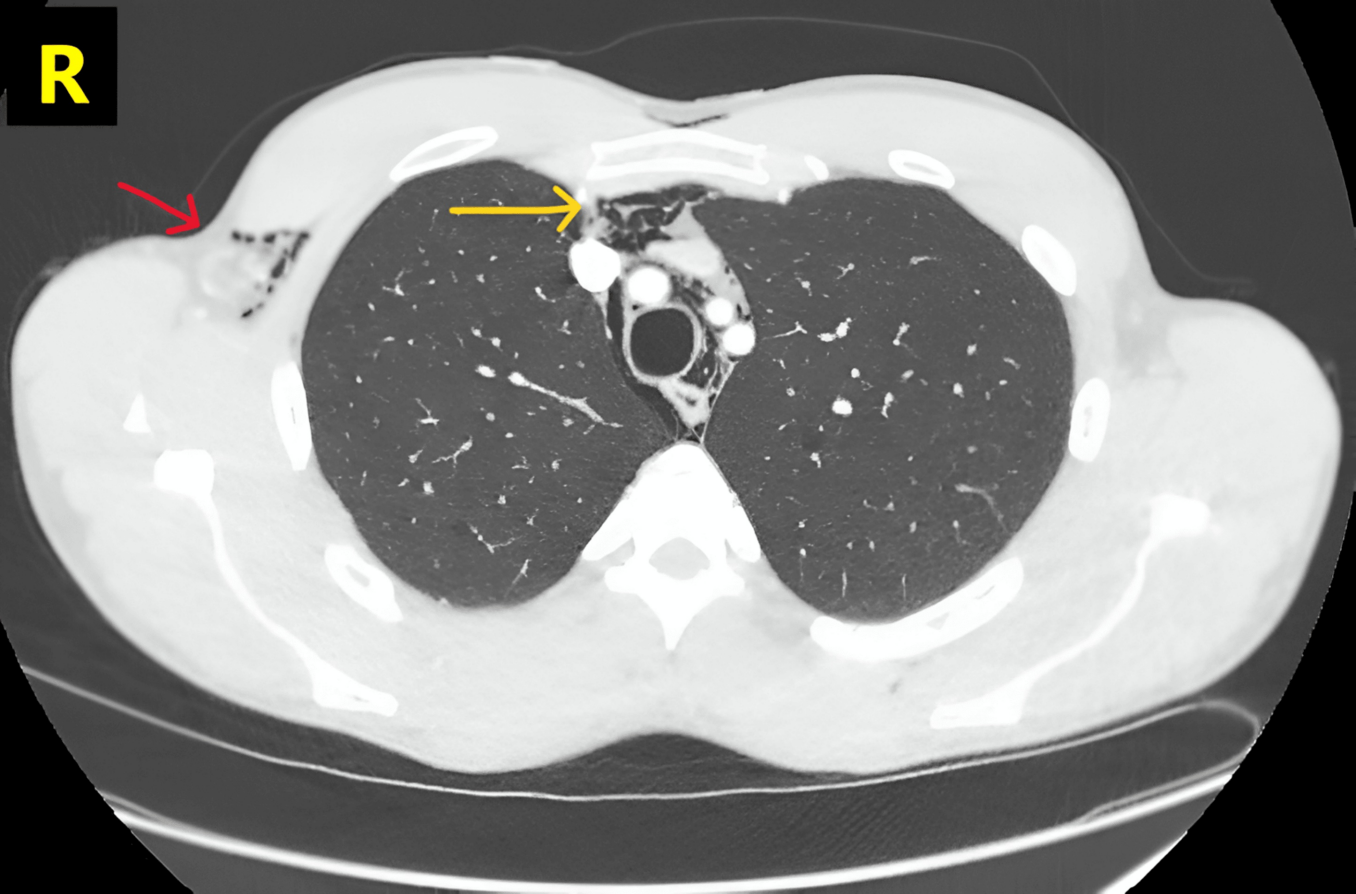
CT of the thorax (shown on lung window) showing pneumomediastinum in the superior mediastinum (yellow arrow), with additional subcutaneous emphysema visible inferolateral to pectoralis major on the right side (red arrow).

On presentation to the emergency department, the patient had a significantly raised WBC count of 22 x 10^9/L, a neutrophil count of 18 x 10^9/L, and a raised CRP of 16 mg/L (Table 1). The rest of the laboratory investigations performed, including liver function tests, renal function tests, venous blood gas (Table 2), and electrolytes, were all within normal ranges. A chest X-ray in the posteroanterior (PA) view (Figure 1) showed the findings of widespread subcutaneous emphysema and pneumomediastinum, visible prominently in the superior mediastinum. The patient went on to have a CT of the thorax with IV contrast, which reported the presence of a small distal oesophageal tear with secondary pneumomediastinum (Figure 2).

**Table 1:** Routine blood investigations conducted in the emergency department on presentation. ALT: alanine aminotransferase; ALP: alkaline phosphatase; INR: international normalized ratio; APTT: activated partial thromboplastin time.

Investigations	Values	Reference range
White cell count	22.5	4-11 x 10*9/L
Neutrophils	18.88	2-7 x 10*9/L
Lymphocytes	1.67	1-3 x 10*9/L
Haemoglobin	148	130-165 g/L
Platelets	255	150-450 x 10*9/L
ALT	32	0-40 U/L
ALP	63	30-130 U/L
Bilirubin	16	0-20 umol/L
Sodium	134	133-145 mmol/L
Potassium	4.4	3.5-5.3 mmol/L
Creatinine	88	62-106 mmol/L
CRP	16	0-4.9 mg/L
Glucose	5.7	3.5-5.5 mmol/L
INR	1.0	0.8-1.2
APTT	27.8	26-38 seconds
Lipase	21	0-60 U/L
Calcium	2.50	2.2-2.6 mmol/L
Magnesium	0.80	0.7-1 mmol/L
Albumin	46	35-50 g/L

**Table 2:** Venous blood gas test done in the emergency department on presentation. pCO2: partial pressure of carbon dioxide; pO2: partial pressure of oxygen.

Venous blood gas	Values	Reference range
pH	7.47	7.35-7.45 mmol/L
pCO2	4.16	4.7-6 kPa
Bicarbonate	22.9	22-29 mmol/L
pO2	6.55	>10.6 kPa
Lactate	1.3	0.6-2.4 mmol/L

Since the patient had a history of excessive vomiting and the chest CT showed pneumomediastinum with suspected oesophageal perforation, the immediate first differential diagnosis was Boerhaave syndrome. The patient was transferred to the emergency department resuscitation bay, and intensive supportive management was initiated, including broad-spectrum IV antibiotics, intravenous fluids, and close monitoring of vital signs [12]. The images were transferred to the local upper GI tertiary centre, which recommended a CT of the chest, abdomen, and pelvis with oral contrast. This demonstrated an isolated pneumomediastinum without any contrast leak indicative of oesophageal perforation.

The initial working diagnosis for the patient, based on the chest X-ray before the report was available, was bilateral small pneumothorax. Although, unlike some patients presenting with Hamman’s syndrome, mistaken as pneumothorax, he was not managed with chest drains due to the small size and the lack of respiratory compromise. The presumed diagnosis then changed to Boerhaave syndrome following a CT of the thorax, which was managed with aggressive supportive measures pending further information. Boerhaave is most commonly seen after excessive vomiting and carries a mortality rate of up to 90% if left untreated [13]. Eventually, the oral contrast study, along with the patient’s clinical picture, and advice from the tertiary upper GI centre supported the diagnosis of spontaneous pneumomediastinum, also known as Hamman’s syndrome.

The patient spent a total of 15 hours in the hospital, all in the emergency department. Initially, when the patient was thought to have Boerhaave syndrome, he was admitted to the resuscitation area for frequent monitoring. He was kept nil by mouth and given intravenous fluids. He received a total of 2 L of Hartmann’s solution during his stay in the emergency department. He also received prophylactic broad-spectrum antibiotics and antifungals, as well as anti-emetics and pain relief.

After observing his clinical status overnight in the emergency department and in light of new imaging results, his diagnosis was changed to Hamman’s syndrome. A trial of a light diet was performed, which he tolerated very well, and IV antibiotics and IV fluids were stopped.

He went on to be discharged from the emergency department with advice to represent should symptoms worsen, new symptoms develop, or any adverse clinical features develop, with no planned follow-up.

## Discussion

Patients with Hamman’s syndrome most commonly present with chest pain and dyspnoea. The most common sign is subcutaneous emphysema [4]. These symptoms overlap with Boerhaave syndrome, which led us to initially treat our patient for presumed oesophageal rupture. One indication from the early stage of the presentation that was in keeping with isolated Hamman’s syndrome as a diagnosis was the normal observations, in keeping with this being a comparatively minor and self-limiting condition [14]. The opposite is true for Boerhaave syndrome, where patients are more likely to be acutely unwell with haemodynamic instability.

We are not unique in failing to diagnose Hamman’s immediately upon presentation. Treharne et al., in their 2021 publication, reported that their initial diagnosis for subcutaneous emphysema was cellulitis [15]. Similarly, it has also been misdiagnosed as an adverse drug reaction in a postpartum patient [16].

Many predisposing factors have been identified that could cause Hamman’s syndrome, such as excessive vomiting and coughing. Interestingly, ethanol intoxication has also been identified as a major causative factor [17]. The pathophysiology for all these causative factors is the same: it is believed that a sudden change in intrathoracic pressure leads to alveolar rupture, causing air to escape into the pulmonary interstitial spaces and ultimately the mediastinum [5].

A plain chest X-ray, both posterior-anterior and lateral views, is considered the investigation of choice for Hamman’s syndrome [18]. In a few cases where the X-ray is inconclusive, a chest CT may be necessary. In our patient, a lateral chest X-ray was not carried out due to high clinical suspicion of Boerhaave syndrome, and therefore we proceeded immediately with a thorax CT with IV contrast.

The management for Hamman’s syndrome is primarily conservative. Historically, prior to a more widespread understanding of the nature and prognosis of the condition, an inpatient stay of one week was typical whilst patients underwent extensive investigations and therapies. However, with increasing clinical confidence in the benign and self-limiting nature of the condition, a much shorter stay and more limited investigations can be required, with the main focus to exclude more serious pathology ahead of a safe discharge home [8].

Bed rest, analgesia, oxygen therapy, and monitoring are considered mainstays of treatment for patients with Hamman’s syndrome. Some patients may benefit from antibiotics if their inflammatory markers are elevated [11]. Oxygen therapy is thought to help absorb free air faster by creating a pressure gradient, resulting in the quicker resolution of pneumomediastinum and subcutaneous emphysema, although the evidence base for this appears weak [5,19].

## Conclusions

In summary, Hamman’s syndrome, although often initially misdiagnosed due to its symptom overlap with more serious conditions like Boerhaave syndrome, is a benign and self-limiting condition. Prompt recognition and appropriate conservative management can lead to excellent outcomes, avoiding unnecessary invasive procedures and prolonged hospital stays.
